# The Accumulation of Health-Promoting Nutrients from Representative Organs across Multiple Developmental Stages in Orange Chinese Cabbage

**DOI:** 10.3390/plants12112120

**Published:** 2023-05-26

**Authors:** Ruixing Zhang, Jiahao Zhang, Chao Li, Qiming Pan, Saeed ul Haq, Walid F. A. Mosa, Fang Fang, Lugang Zhang, Baohua Li

**Affiliations:** 1 State Key Laboratory of Crop Stress Biology for Arid Areas, College of Horticulture, Northwest A&F University, Yangling, Xianyang 712100, China; xingqing@nwafu.edu.cn (R.Z.); zjh_nwafu@nwafu.edu.cn (J.Z.); lc1999@nwsuaf.edu.cn (C.L.); 2020055340@nwsuaf.edu.cn (Q.P.); 2022205044@mail.nwpu.edu.cn (F.F.); lugangzh@nwafu.edu.cn (L.Z.); 2 Department of Horticulture, University of Agriculture Peshawar, Peshawar 25120, Pakistan; haq@aup.edu.pk; 3 Plant Production Department (Horticulture-Pomology), Faculty of Agriculture (Saba Basha), Alexandria University, Alexandria 21531, Egypt; walidbreeder@yahoo.com

**Keywords:** orange Chinese cabbage, indolic glucosinolate, carotenoids, nutrition, developmental stage, dietary instruction

## Abstract

Orange Chinese cabbage (*Brassica rapa* L. ssp. *pekinensis*) is an excellent source of health-promoting nutrients that could reduce the risk of chronic diseases. This study mainly investigated the accumulation patterns of eight lines of orange Chinese cabbage for indolic glucosinolates (GLSs) and pigment content from representative plant organs across multiple developmental stages. The indolic GLSs were highly accumulated at the rosette stage (S2), especially in inner and middle leaves, and the order of indolic GLSs accumulation in non-edible organs was flower > seed > stem > silique. The expression levels of biosynthetic genes in light signaling, MEP, carotenoids, and GLS pathways were consistent with the metabolic accumulation patterns. The results of a principal component analysis show a clear separation of high indolic GLS lines (15S1094 and 18BC6) from low indolic GLS lines (20S530). A negative correlation between the accumulation of indolic GLS and carotenoids was identified in our study. Our work contributes to providing valuable knowledge required to breed, grow, and select orange Chinese cabbage varieties and their eatable organs with higher nutritional value.

## 1. Introduction

Glucosinolates (GLSs) are a large group of plant secondary metabolites of cruciferous vegetables, and they play crucial roles in plants’ responses to biotic and abiotic stresses and contribute to the unique taste, flavor, and nutrients of cruciferous vegetables [1,2,3]. It is generally accepted that the GLS metabolic pathway evolved from the metabolism of cyanoglucoside, which is a relatively conserved secondary metabolite in higher plants, both of which use amino acids as precursors [4,5]. GLSs could be divided into indolic GLSs (side chains derived from tryptophan), aliphatic GLSs (side chains derived from methionine, valine, alanine, isoleucine, and leucine), and aromatic GLSs (side chains derived from tyrosine and phenylalanine) [6,7]. The distribution and content of phytochemicals in plants are often affected by genetic, developmental, and environmental factors [8,9,10]. Among these factors, genotypic variation is generally considered the dominant factor in shaping GLS accumulation [11]. Since GLSs are important defense metabolites of cruciferous plants [12], it is also crucial to study the distribution and accumulation of GLSs in representative organs and leaf positions of Chinese cabbage (*Brassica rapa* L. ssp. *pekinensis*) to evaluate the dynamics of plant defense mechanisms; this would also provide valuable information of the nutritional value of different organs across the multiple growth stages in Chinese cabbage.

Cruciferous crops, especially Chinese cabbage, are rich in natural bioactive substances such as GLSs, carotenoids, and phenolic compounds [13,14,15], while cruciferous crops with different genetic backgrounds often contain different types of GLSs, as previously reported in broccoli [16], kale [17], cauliflower [18], white radish [19], and other cruciferous vegetables. The content of GLSs accounts for around 1–10% of the dry weight in Brassica vegetables [20]. In addition, GLSs and their degradation products undergo transformation, assimilation, absorption, and elimination after being ingested in the human gut, and daily intake of a certain amount of cruciferous plants can significantly reduce the risks of multiple cancers [21,22]; this potential benefit is mainly due to the presence of degradation products, isothiocyanates, which have anti-inflammatory properties [23] and induce apoptosis in mutant cells including indole-3-carbinol (I3C), the breakdown product of indol-3-ylmethyl (I3M). I3C can inhibit various cancers, including prostate cancer [24,25], and was reported to induce phase 2 enzymes, which are involved in the detoxification of intracellular compounds [26]. I3C was also shown to have pleiotropic protective effects on hepatic steatosis, hepatic cirrhosis, and hepatocellular carcinoma injuries [24]. I3C and its major in vivo product, 3,3′-diindolylmethane (DIM), are effective cancer chemopreventive agents [27]. In addition to indolic GLSs, orange Chinese cabbage is also rich in flavonoids, carotenoids, and other biologically active compounds [28]. Therefore, the relationship between these key nutritional metabolites, especially between the distribution of indolic GLSs in orange Chinese cabbage and their beneficial effects on human dietary health, needs to be further explored.

GLS accumulation among various tissues is crucial for understanding the changes in their transport from source to sink [29,30], as GLSs could be transported from leaf sites to roots and inflorescence [31]. In *Arabidopsis thaliana* (L.) Heynh., both rosette leaves (or inflorescences) and roots can synthesize indolic GLSs [32]. The total GLS content of different tissues in Chinese cabbage was identified in the following order: seed > flower > young leaves > stem > root [33]. Total GLS content was found to be greater in turnip compared with cauliflower, followed by white-headed cabbage sprouts [34]. However, studies on the indolic GLSs of non-edible organs of orange Chinese cabbage have not yet been reported.

The nutritional value of the edible organs of Chinese cabbage is receiving increasing research attention [18,35]. Previous studies have primarily focused on the carotenoid metabolism pathway of orange Chinese cabbage [36,37], in which the *Br-or* gene was cloned and found to regulate carotenoid metabolism [38]. However, the key nutritional metabolites in Brassica vegetables, including indolic GLSs, have not yet been systemically studied. Therefore, the current study aimed to measure multiple lines of orange Chinese cabbages for their accumulation of indolic GLSs and carotenoids in representative plant organs across multiple growth stages, and to explore the delicate balance between the metabolic biosynthesis among the major nutrients in orange Chinese cabbage.

## 2. Results

### 2.1. The Agronomic Traits of Selected Orange Chinese Cabbage Lines

**Figure 1:**
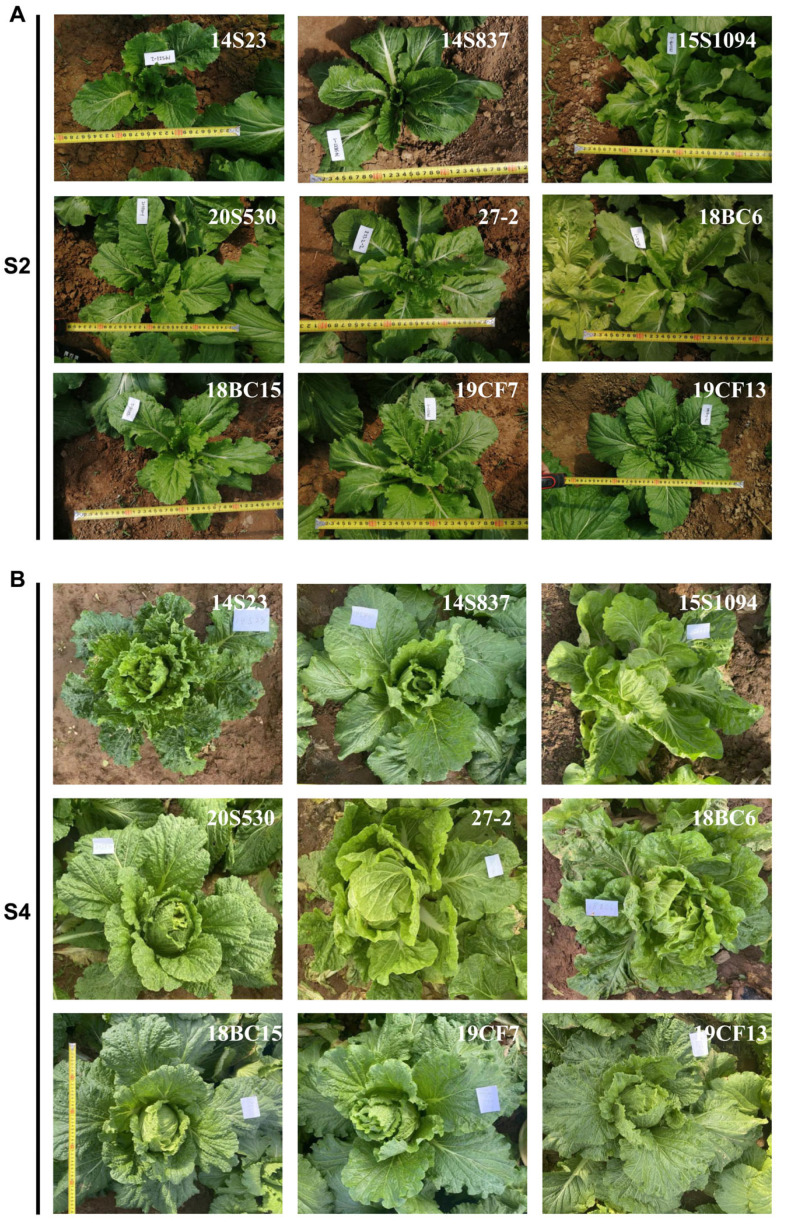
Phenotypes of the orange Chinese cabbage at rosette stage and mid-head stage. (**A**) Rosette phenotype (S2). (**B**) Metaphase phenotype (S4).

**Figure 2:**
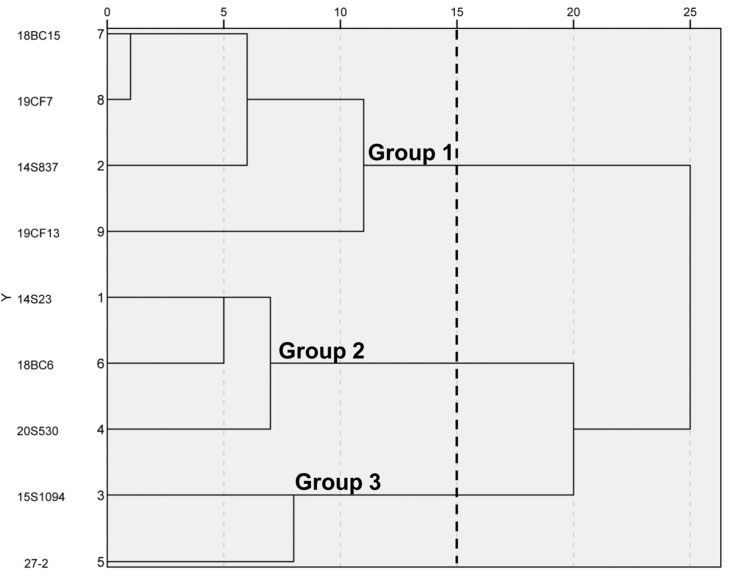
Clustering diagram of 9 Chinese cabbage lines by the shapes of the leaf head. Phenotypic relationships between the lines were derived from the Euclidean distance (ED). The 9 lines were divided into 3 clusters.

To select representative orange Chinese cabbage lines, five inbred lines and three hybrid lines with different breeding backgrounds were used for this study, together with common white Chinese cabbage line 14S23 as the control (Figure 1). By leaf head, these lines could be divided into three categories, 14S23 as kidney-shaped, 15S1094, 27-2, and 18BC6 as round, and the rest as long and smooth. Detailed agronomic traits of these nine lines are presented in Table S1. SPSS 23.0 was used to standardize the data, and the Wald method was used to perform cluster analysis on the nine Chinese cabbage lines according to agronomic traits. As shown in Figure 2, when the Euclidean distance is fifteen, the nine lines are divided into three groups, among which, Group 1 includes 18BC15, 19CF7, 14S837, and19CF13; Group 2 includes 14S23, 18BC6, and 20S530; and Group 3 includes 15S1094 and 27-2. These results demonstrate the significant phenotypic differences in the agronomic traits of the selected nine lines.

### 2.2. Pigment Content and Antioxidant Activity

**Figure 3:**
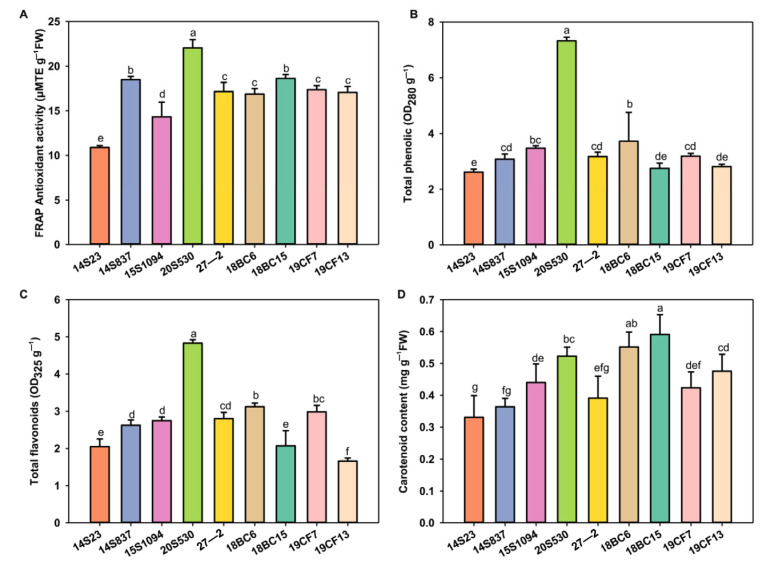
The antioxidant activity and pigment content of the orange Chinese cabbage. (**A**–**D**) Antioxidant activity, and total phenolic, flavonoid, and carotenoid contents of the orange Chinese cabbage leaf head. The data are shown as the mean of three independent replicates (mean ± SE). Small letters indicate significant differences at *p* < 0.05, respectively.

Leaf color is one of the most important agronomic traits of Chinese cabbage, and the selected lines showed wide differences for this trait. To this end, we measured the pigment content of leaf head samples of these lines, together with the antioxidant activities in these lines (Figure 3). The selected lines showed variations for both of these two traits. Specifically, ferric-reducing antioxidant power (FRAP), total phenols, and flavonoids were enriched in 20S530 and 18BC6, while carotenoid content was enriched in 18BC15 and 18BC6. The inbred lines of 20S530, 18BC15, and 18BC6 with high antioxidant activity could potentially be selected as pigment-enriched Chinese cabbage candidate lines for breeding efforts.

### 2.3. Dynamic Accumulation of Indolic GLSs across Vegetative Growth Stages

**Figure 4:**
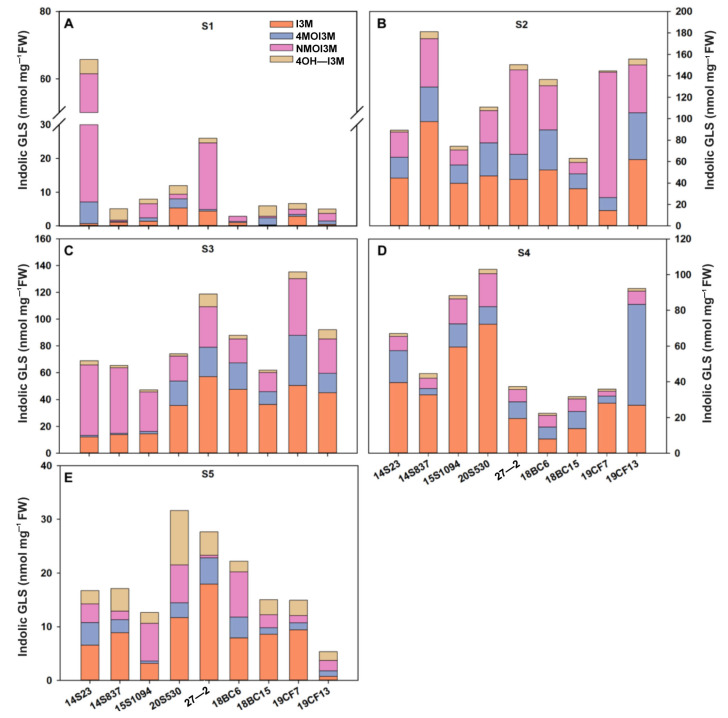
The content of indolic GLSs in the outermost leaves of the orange Chinese cabbage lines at multiple developmental stages (S1, S2, S3, S4, and S5). (**A**) The content of indolic GLSs of the Chinese cabbage lines at S1 stages. (**B**) The content of indolic GLSs of the Chinese cabbage lines at S2 stages. (**C**) The content of indolic GLSs of the Chinese cabbage lines at S3 stages. (**D**) The content of indolic GLSs of the Chinese cabbage lines at S4 stages. (**E**) The content of indolic GLSs of the Chinese cabbage lines at S5 stages. I3M (indol-3-ylmethyl), 4MO-I3M (4-methoxy-indol-3-ylmethyl), NMO-I3M (N-methoxy-indol-3-ylmethyl), and 4OH-I3M (4-hydroxy-indol-3-ylmethyl).

GLSs are one of the major secondary metabolites in Chinese cabbages, and some serve as key nutrients. Here, we systemically studied GLS accumulation in the selected lines. We found that the major class of GLSs in our tested orange Chinese cabbage lines are indolic GLSs, and they were consistently detected in all tested tissues and growth stages; thus, we focused on the indolic GLSs in this study. Firstly, we evaluated indolic GLSs at different developmental stages as shown in Figure 4. Overall, the total indolic GLS content gradually increased and then decreased across the developmental stages. Specifically, at the S1 stage, the indolic GLS content of 14S23 was the highest, reaching 65.77 nmol/mg FW (Figure 4A). With the growth of the plants, the large variations between high and low indolic GLSs become smaller (Figure 4B–D). At the stage of commercial maturity (S4), the indolic GLS content was high in 15S1094, 20S530, and 19CF13 (Figure 4D). It is worth noting that in the S5 stage, the content of GLSs in most orange Chinese cabbage lines decreased. In addition, the content of I3M gradually increased and then decreased during the vegetative growth stages. These results clearly showed the high dynamics of indolic GLS accumulation across growth stages, and 15S1094 with high I3M content at commercial maturity (S4) could potentially be used as important orange Chinese cabbage lines with high GLS accumulation in future breeding efforts.

### 2.4. The Accumulation of Indolic GLSs in Representative Tissues

**Figure 5:**
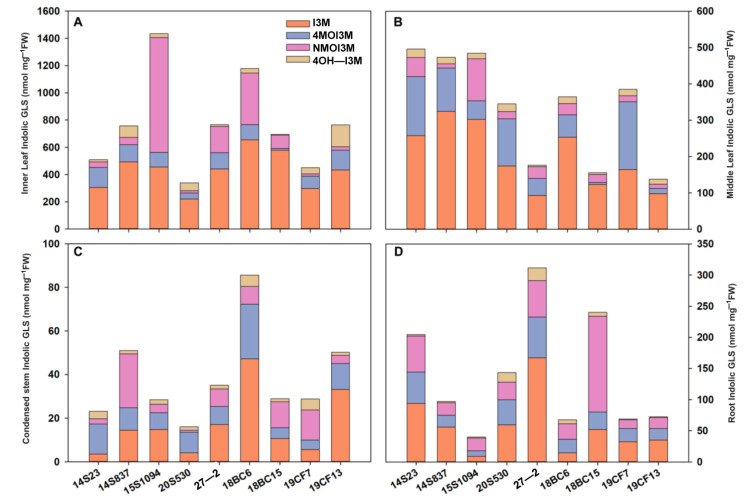
The indolic GLS content of leaf (including inner leaf and middle leaf), condensed stem, and root of the 9 lines of Chinese cabbage in the S4 period. (**A**) The indolic GLS content of inner leaf in the S4 period. (**B**) The indolic GLS content of middle leaf in the S4 period. (**C**) The indolic GLS content of condensed stem in the S4 period. (**D**) The indolic GLS content of root in the S4 period.

The order of GLS content in the tissues of orange Chinese cabbage was identified as follows: inner leaf > middle leaf > root > condensed stem (Figure 5), and the content of I3M in the inner and middle leaves was higher than that in the condensed stem and root. In inner leaves, the indolic GLS content in 15S1094 was higher, reaching 1616.56 nmol/mg FW. The indolic GLS content in 20S530 was lower, reaching 338.95 and 509.03 nmol/mg FW, and I3M reaching 10.56% and 9.60%, respectively (Figure 5A and Figure S1). In the middle part, 18BC6 had a higher accumulation level, while 27-2 showed a lower level of GLS accumulation (Figure 5B). Notably, there was a similar accumulation pattern in the condensed stem as in the inner lobe (Figure 5C). The indolic GLSs in roots of 18BC6 and 15S1094 reached 67.49 and 39.74 nmol/mg FW, respectively, and the GLS accumulation patterns were significantly different in the leaves (Figure 5D). These results suggest that different parts of orange Chinese cabbage have large variations in GLS accumulation. At the same time, as the main edible parts, consumers are advised to choose 15S1094 and 18BC6 with high GLS content for consumption.

**Figure 6:**
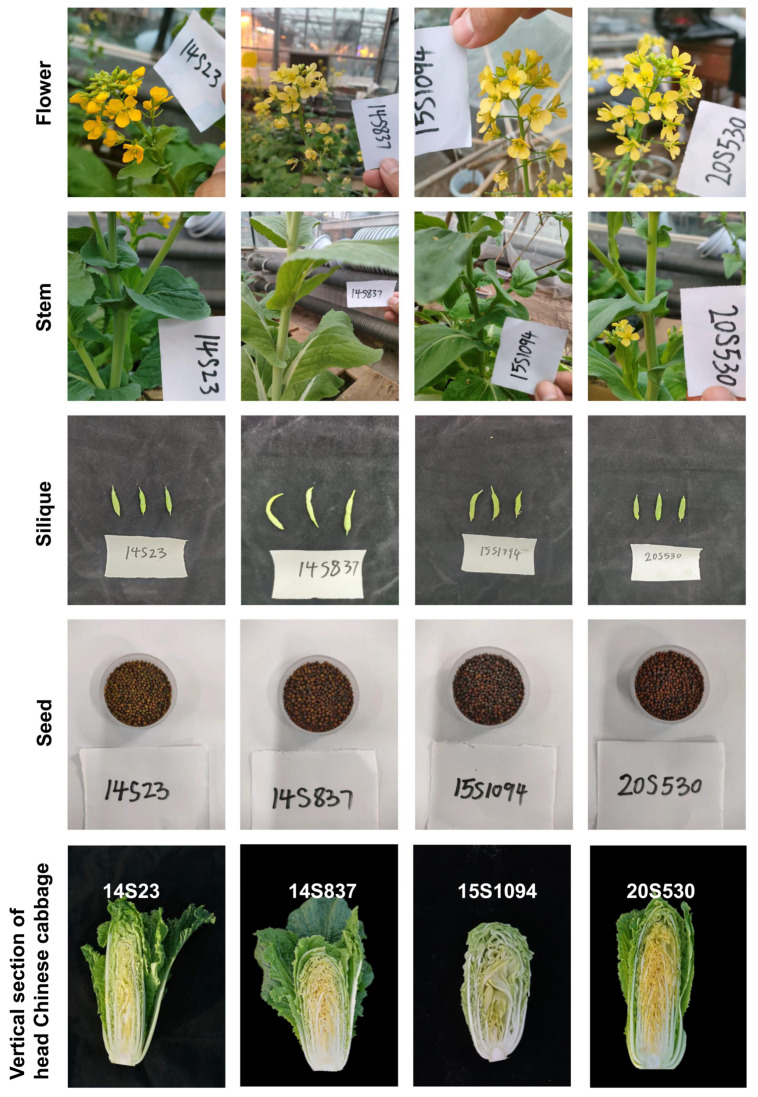
The phenotypes of flowers, stems, siliques, seeds, and longitudinal sections of leaf heads in orange Chinese cabbage lines 14S837, 15S1094, and 20S530 together with the common line 14S23 as the control.

**Figure 7:**
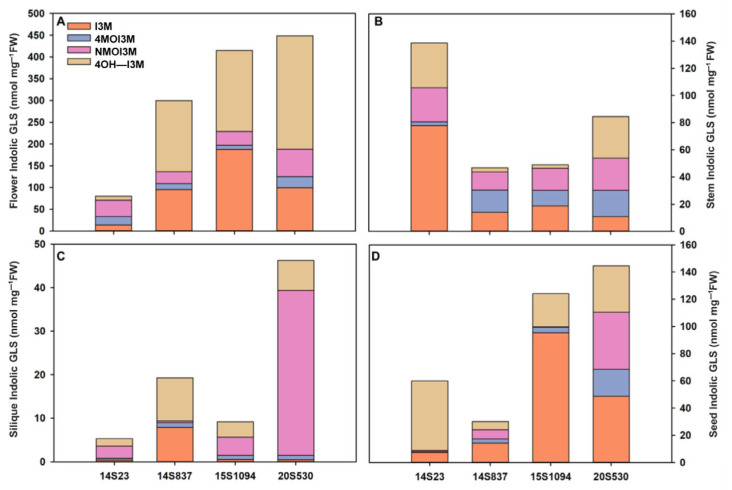
Indolic GLS content in representative organs (flowers, stems, siliques, and seeds) of orange Chinese cabbage (14S837, 15S1094, and 20S530) lines. (**A**) The indolic GLS content of flowers in orange Chinese cabbage lines. (**B**) The indolic GLS content of stems in orange Chinese cabbage lines. (**C**) The indolic GLS content of siliques in orange Chinese cabbage lines. (**D**) The indolic GLS content of seeds in orange Chinese cabbage lines.

Furthermore, we also selected orange Chinese cabbage (14S837, 15S1094, and 20S530) for their indolic GLS content in reproductive growth stages (flowers, stems, siliques, and seeds) (Figure 6 and Figure 7). The indolic GLS content in floral organs and seeds was higher than that in stems and siliques (Figure 7). Specifically, the indolic GLS I3M content of control line 14S23 with dark orange flowers was lower than that in the cabbage lines with light yellow flowers (14S837, 15S1094, and 20S530). However, there is an opposite pattern of accumulation in the 14S23 stem (Figure 7A). I3M content in siliques is extremely low, and in seeds, I3M content in 14S23 is lower than that in 15S1094 and 20S530 (Figure 7C,D). The diverse patterns of tissue indolic GLS accumulation offer the potential for enhanced human nutrition in multiple tissues other than leaf tissue.

### 2.5. The Interconnection of the Nutrient Accumulation in Orange Chinese Cabbage Lines

**Figure 8:**
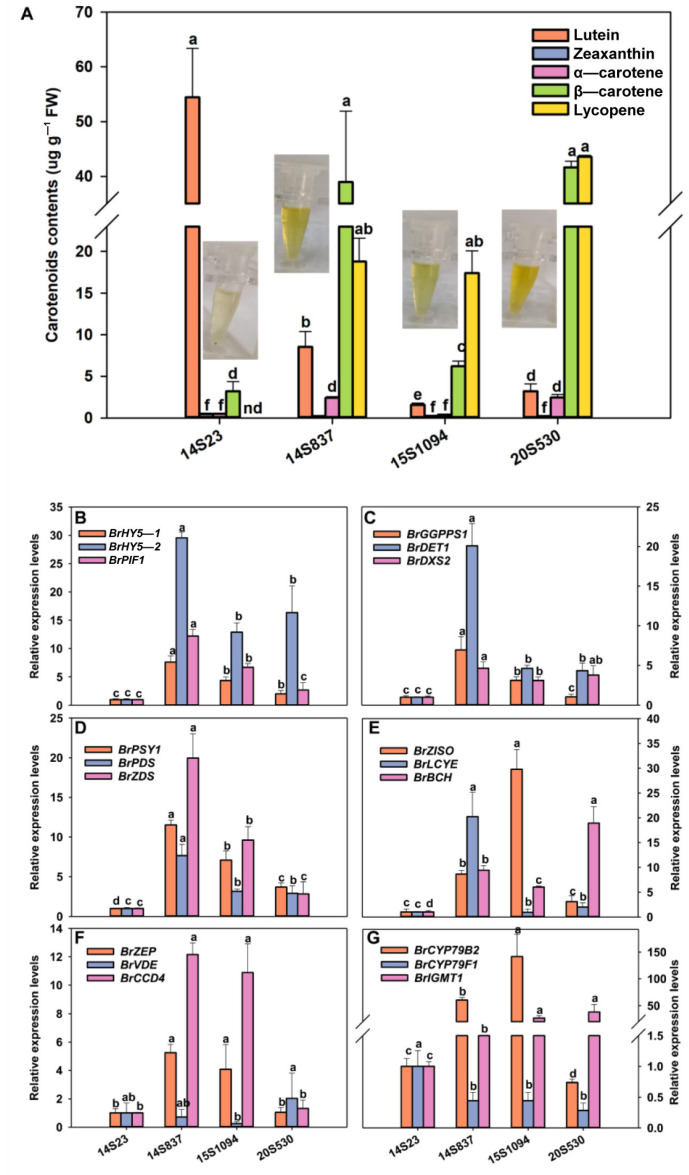
Carotenoid content in orange Chinese cabbage lines. (**A**) Carotenoid content, (**B**) light-signal-related gene expression levels (*BrHY5-1*, *BrHY5-2*, and *BrPIF1*). (**C**) MEP-pathway-related gene expression levels (*BrGGPPS1*, *BrDET1*, and *BrDXS2*). (**D**–**F**) Expression levels of carotenoid metabolism-related genes (*BrPSY1*, *BrPDS*, *BrZDS*, *BrZISO*, *BrLCYE*, *BrBCH*, *BrZEP*, *BrVDE*, and *BrCCD4*). (**G**) The expression levels of indolic GLS metabolism-related genes (*BrCYP79B2*, *BrCYP79F1*, and *BrIGMT1*). Mean values and SEs for three replicates are shown. Lowercase letters represent significance level *p* < 0.05.

We further explored the relationship between the accumulation of indolic GLS and carotenoid components in Chinese cabbage (Figure 6, Figure 7 and Figure 8). Specifically, the line with the highest content of zeaxanthin, α-carotene, β-carotene, and lycopene was 20S530, followed by 14S837, 15S1094, and 14S23 (Figure 8A). We found that the expression levels of light signal genes *BrHY5-1*, *BrHY5-2*, *BrPIF1*, MEP metabolic pathway genes *BrGGPPS1*, *BrDET1*, and *BrDXS2* were significantly higher in 14S837 and 15S1094 than those in 14S23 (Figure 8B). Overall, carotenoid metabolism genes in 20S530 and 15S094 had higher transcript levels than those in 14S23 (Figure 8D–E). In addition, the transcription levels of indolic GLS metabolic genes *BrCYP79B2* and *BrIGMT1* in 20S530 and 15S094 were significantly higher than those in 14S23 (Figure 8G). These results show that the carotenoid components of Chinese cabbage with diverse head colors are different, and the expression levels of genes in the light signal, the MEP metabolic pathway, and the carotenoid and indolic GLS metabolic pathways were higher in the high carotenoid 20S530 and 15S1094 lines than those in low carotenoid 14S23 lines, suggesting the complex interconnections of these metabolic pathways in Chinese Cabbage lines.

**Figure 9:**
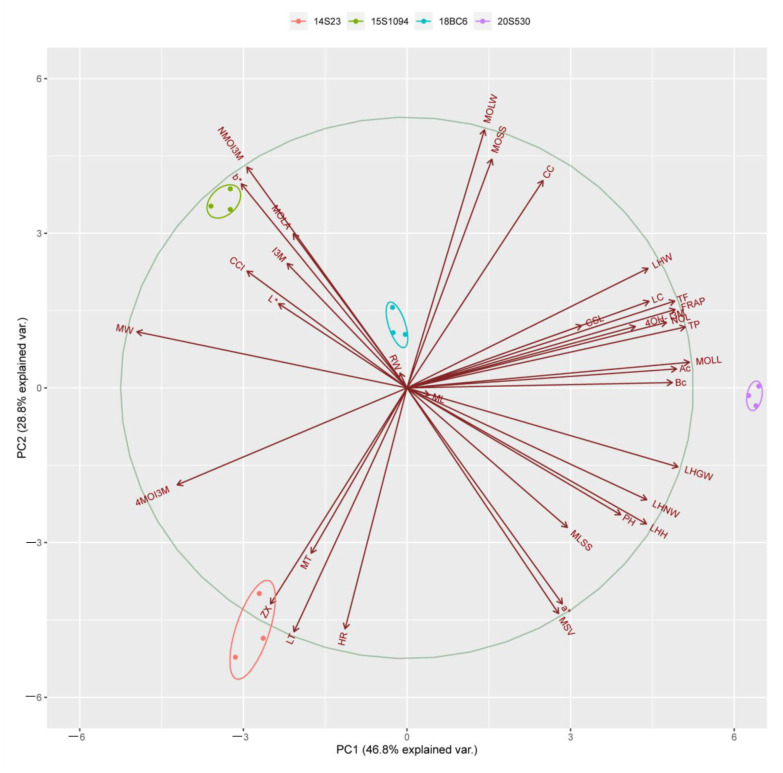
The PCA scores of different Chinese cabbage (14S23, 20S530, 15S1094, and 18BC6) biological traits, pigments, and GLS. The axes PC1 and PC2 in the figure are the first and second principal components (i.e., the interpretation rate of latent variables to the difference); dots represent samples, and different colors represent different groups; ellipses represent the core area added to the groups according to the 68% confidence interval, which is convenient to observe whether the groups are separated; the arrows represent the original variables, and their direction represents the correlation between the original variables and the principal components; and the length represents the contribution of the original data to the principal components. Total flavonoid content (TF), total phenolic (TP), carotenoid content (CC), lutein (LT), zeaxanthin (ZX), α-carotene (Ac), β-carotene (Bc), and Lycopene (LC).

Principal component analysis (PCA) was performed to identify differences between the high indolic GLS lines (18BC6 and 15S1094) and the low indolic GLS lines (14S23 and 20S530). The 14S23, 20S530, 18BC6, and 15S1094 lines can be well differentiated along the PC1 level. The percent variance obtained for all principal components (PCs) is shown in Table S2. The first component (PC1) and the second component (PC2) explained 46.80% and 28.80% of the variance, respectively (Figure 9). Subsequently, we performed a correlation analysis on 15S1094 and 20S530, which were significantly different (Figure S2). Among them, the NMOI3M was negatively correlated between TP and TF in 15S1094, the I3M was negatively correlated with LC. In 20S530, the Bc was negatively correlated with HR, 4OH-I3M. Meanwhile, the NMOI3M was negatively correlated with the MOLW in the 20S530 line. Overall, the mainly indolic GLSs, carotenoids, and biological traits were negatively correlated in the 15S1094 and 20S530 lines.

## 3. Discussion

Chinese cabbage leaf head is an important edible organ with important nutritional and therapeutic values, and orange Chinese cabbage is highly popular due to its higher carotenoid and flavonoid contents [36,37], while indolic GLS accumulation of the orange Chinese cabbage has not yet been investigated [28]. The main purpose of this study is to investigate the distribution of indolic GLSs in representative organs of orange Chinese cabbage across multiple growth stages, and the interconnection between the content of major nutrients and major agronomic traits, as well as the trade-offs in GLS and carotenoid metabolism. Our work provides clues for identifying high indolic GLSs in orange Chinese cabbage lines with higher nutritional value for future breeding efforts.

### 3.1. Antioxidant Pigment Concentrations in Multiple Growth Stages

Plant pigments are involved in photomorphogenesis and defense against abiotic stresses for maximum ecological fitness [39]. In this study, the morphological and biological phenotypes of orange Chinese cabbage lines were quite different (Figure 1) and were categorized into three distinct groups based on these traits (Figure 2). This study focused on 20S530 (Group 2) and 15S1094 (Group 3), and the photosynthetic pigment, antioxidant activity, and TF, TP, and MSV contents in 20S530 were significantly higher than those in 15S1094 (Figure 3 and Table S2). The carbon double bond structure of TP and TF contributes to its strong antioxidant capacity [40], which can scavenge singlet oxygen (^1^O_2_). Previous studies also reported that flavonoids and total phenols in Chinese cabbage stabilize pigments and exhibit antioxidant properties [41]. Furthermore, chlorophyll serves as an effective antioxidant [42], which can enhance plant defense response. These results indicate that 20S530 may use pigment secondary metabolites to achieve environmental adaptation, while 15S1094 may utilize other secondary metabolites for the adaption.

### 3.2. Indolic GLS Accumulation in Representative Tissues and Leaf Positions

GLSs have been intensively studied as an important plant defense compound [2,43] and they require about 15% of the photosynthetic energy [44]. Here, we studied indolic GLS content in different periods of vegetative growth of orange Chinese cabbage lines. In general, the GLSs first increased (S1, S2, and S3) and then decreased (S4 and S5) during the growth process of Chinese cabbage (Figure 4), which was likely due to the degradation and relocation of GLSs in the late growth stage, as reported in the GLS accumulation pattern of senescent outer leaves in Arabidopsis [45] and the GLS accumulation pattern of inner leaves of Chinese cabbage [46]. Furthermore, the reduced accumulation of defensive compounds in the older leaves may also be due to the passive diffusion of chemicals as the leaves age and expand [47], and this helps explain the decreased levels of the defensive compound GLS.

In this study, three lines of orange Chinese cabbage with large morphological differences together with a common control line were further chosen to investigate the indolic GLS content in representative organs, and the concentration of indolic GLSs was determined as follows: flowers > seeds > stems > siliques (Figure 7). Kim, et al. [33] also found that the highest GLS content in the aerial section was in flowers, followed by young leaves and stems. Notably, control line 14S23 inflorescences had high pigment content, but low GLS content, which likely explains the delicate balance between the biosynthesis of the energy-rich metabolites of pigments and GLSs [48]. It has been reported that GLSs are mainly transported through the stem [49], indicating a source–sink relationship in the GLS accumulation process. The high level of GLS accumulation in the stem tissue of 14S23 points to the potential utilization of by-products (such as stem) as food additives to improve food nutritional quality and to fully use nutrients in Chinese cabbage.

### 3.3. The Selection of a High GLS Line of Orange Chinese Cabbage to Promote Human Diet Health

The leaf head of Chinese cabbage is the most important and nutritious part of this vegetable crop [43,50]. Plants exhibit predictable GLS changes at different scales, which undergo convergent evolution with herbivores to maximize ecological adaptability. This study further focused on the accumulation pattern of indolic GLSs at different leaf positions (Figure 5). Specifically, most cabbage inner leaves contain a large amount of I3M. The consumption of indolic I3M can suppress multiple cancers [51,52]; this potential benefit is mainly due to the formation of cell mutagenesis protector I3C as a degradation product of I3M [53]. From a plant defense perspective, NMOI3M has stronger effects against aphids in the *Arabidopsis* [54,55]. The order of GLS accumulation in tissues of Chinese cabbage was identified as follows: leaf > middle leaf > outer leaf. This is in line with the optimal defense theory as the most vulnerable organ has the highest level of defense compounds [56]. The inner leaves of Arabidopsis rosettes contain more GLSs than the outer leaves [30], and Zhao et al. [43] also reported that the content of GLSs in the inner leaves of cabbage is significantly higher than that in the outer leaves. This suggests that consumers and breeders should select 15S1094 as a high GLS Chinese cabbage line and take its inner young leaves as the top choice for food.

This study also evaluated the accumulation pattern of indolic GLSs in the condensed stem and roots. Among them, the GLS accumulation pattern in the condensed stem of 15S1094 and 20S530 was similar to those in the inner leaves and most of the non-edible organs (Figure 5C,D). Despite being an important nutrient transport hub, the high concentration of GLSs in the condensed stem has received little attention. Similarly, previous reports have highlighted the utilization of roots, as observed in cabbage stems [18]. The accumulation of GLSs in roots is a complex process that involves a long-distance transport mechanism coordinated by GLS transporters, such as GTR [57], and it has also been suggested that GLSs can be transported in both shoots and underground [32]; the specific complex mechanism requires further study.

### 3.4. Energy Trade-Offs in GLS and Carotenoid Metabolism

Orange Chinese cabbage also is obviously rich in carotenoid accumulation (Figure 8), particularly the carotenoid components lycopene and β-carotene. The contents of lycopene and β-carotene in 20S530 were significantly higher than those in 15S1094 at the S4 period, while a high level of lutein and no lycopene were detected in 14S23. The gene expression study of the light signal, MEP metabolism, carotenoid metabolism, and GLS metabolism pathways were consistent with the metabolic accumulation pattern of the corresponding metabolites, except for *BrVDE* and *BrCYP79F1* (Figure 8). *VDE* is involved in the xanthophyll cycle and *CYP79F1* participates in GLS metabolism [58,59], both of which were highly expressed in 14S23 lines, further triggering the accumulation of lutein. Moreover, this study revealed a negative correlation between carotenoid and GLS fractions (Figure S2). Previous reports highlighted the high cost of metabolizing secondary metabolites such as GLSs and terpenoids (carotenoids) [44,60], supporting the negative correlation between growth and high metabolism [61], as reported in *Arabidopsis* [62] and *Plantago lanceolata* [63]. Additionally, there is also a close relationship between phenylpropane metabolism and isoprene metabolism [64], showing the delicate and complex interconnections of plant metabolic pathways [47].

## 4. Materials and Methods

### 4.1. Plant Materials and Growth Conditions

**Figure 10:**
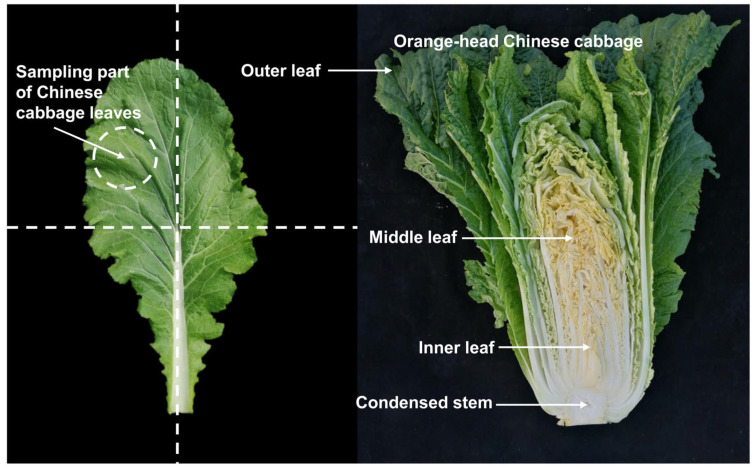
Representative photographs of Chinese cabbage sampling locations.

Eight representative lines of orange Chinese cabbage were selected and used in this study, including 14S837, 15S1094, 20S530, 18BC, and 18BC15 as inbred lines and 19CF7, 19CF13, and Z27-2 as hybrid lines, together with the common Chinese cabbage inbred line 14S23 as the control. The seeds of the above lines were sown in 50-hole plug trays on August 1, 2021, and grown on the experimental farm of Northwest A&F University (Longitude: 108°10′ E, Latitude: 34°31′ N). The lines were established in a randomized block design with 3 replicates and 15 plants. The plot area was about 8.5 mm^2^, the row spacing was 0.9 m, and the plant spacing within the row was 0.6 m. Field fertilizers, weeding, and water management were conducted using standard agricultural practices. The vegetative growth period is five leaves and one heart; the rosette stage, early stage, middle stage, and end stage are represented by S1, S2, S3, S4, and S5, respectively. The sampling times were 15 August 2021, 15 September 2021, 18 October 2021, 12 November 2021, and 2 December 2021. From 9:00 am to 10:00 am for each sampling, three mature and healthy Chinese cabbage heads of uniform size were cut into three parts, wrapped in tin foil, and immediately frozen in liquid nitrogen and stored at −80 °C until analysis. The specific sampling site is depicted in Figure 10.

### 4.2. Determination of Leaf Color

The leaf color analysis of Chinese cabbage was conducted following the method described by Zhou et al. [65]. The measurements of L*, a*, and b* values were performed using a CR-410 Chromo meter (CR 410 Chromo meter, Japan). A positive value of the luminance factor L* indicates orange, and a negative L* indicates white. Positive a* values represent red to purple, while negative a* values represent cyan. Positive b* values indicate yellow, while negative b* values indicate blue.

### 4.3. Investigation of Biological Traits

The agronomic traits were determined for the investigation according to Liu et al. [66], and the main agronomic indicators include: maximum outer leaf soluble solids (MOSS), middle leaf soluble solids (MLSS), number of outer leaves (NOL), plant height (PH), maximum outer leaf width (MOLW), maximum outer leaf length (MOLL), maximum outer leaf area (MOLA), maximum outer leaf SPAD value (MSV), leaf head gross weight (LHGW), leaf head net weight (LHNW), root weight (RW), midrib length (ML), midrib width (MW), midrib thickness (MT), condensed stem length (CSL), leaf head height (LHH), leaf head width (LHW), and head length/head width ratio (HR). At the same time, referring to the method of Badu-Apraku et al. [67], ward minimum variance cluster analysis was performed on agronomic trait indicators. The soluble solid content was determined using a PAL-1 digital sugar meter (ATAGO Corporation, Tokyo, Japan). The chlorophyll content of Chinese cabbage was determined using a portable hand-held chlorophyll meter SPAD-502 (Konica-Minolta, Japan, SPAD-502). The Chinese cabbage leaf area was measured using a portable leaf area meter (Yaxin-1242, Beijing, China). All assays were conducted with at least three biological replicates.

### 4.4. Analysis of Total Carotenoids, Total Phenols, and Flavonoids

The determination of total carotenoids was carried out according to the method of [68]. An amount of 0.2 g of fresh Chinese cabbage sample was immersed in 25 mL of 96% ethanol, extracted at 4 °C for 12 h, and then centrifuged at 8000 rpm/15min, and the supernatant was measured at 665 nm, 649 nm, and 470 nm according to the calculation formula method described by Deng et al. [69]. The content of pigment was calculated using the following equations: Chlorophyll a (Chl a) (mg/L) = 13.95 A665 − 6.88 A649; Chlorophyll b (Chlb) (mg/L) = 24.96 A649 − 7.32 A665; and Carotenoids (Car) (mg/L) = (1000 A470 − 2.05 Chla − 114.8 Chlb)/245. Total phenols (TPs) and flavonoids (TFs) were determined using the method of Huang et al. [70], with slight modifications. Briefly, the 0.5 g of fresh Chinese cabbage sample was ground with 1% (*v/v*) hydrochloric acid and methanol at 4 °C, and then diluted to 20 mL, stored in the dark for 30 min, and then centrifuged at 12,000 rpm/15 min. Then, we aspirated the supernatant, and the absorbance of TPs and TFs was measured at 280 nm and 325 nm using an ultraviolet spectrophotometer (UV-1800, Shimadzu, Japan), respectively. The contents of TPs and TFs are shown as OD_280_/g FW and OD325/g FW, respectively.

### 4.5. Measurement of Total Antioxidant Ability

FRAP assays were carried out according to Thaipong et al. [71], with minor modifications. In short, 0.5 g of fresh Chinese cabbage tissue was weighed, and the volume was made up to 10 mL with absolute ethanol. After leaching for 30 min, the solution was centrifuged at 4500 rpm for 15 min, and the supernatant was collected and stored in the dark. An amount of 200 mL of the supernatant was then added to 2.8 mL of FRAP solution (0.3 mol/L acetate buffer, 10 mmol/L tripyridyltriazine solution, 20 mmol/L FeCl_3_ solution, v:v:v, 10:1:1) and reacted for 1 h at 37 °C under constant temperature conditions, and the absorbance at 596 nm was measured using a UV-1800 spectrophotometer. The calculation formula was carried out according to He et al. [72]. Methanol was used to prepare 10 μM, 20 μM, 40 μM, 70 μM, 150 μM, and 200 μM Trolox solutions, and the absorbance measured at 596 nm was used as the ordinate to obtain the standard curve (y = 16.1290x − 0.0003, R^2^ = 0.99). The FRAP was expressed as the Trolox equivalent antioxidant capacity (TEAC, μM TE g^–1^ fresh weight).

### 4.6. Sample Pretreatment, Extraction, and Analysis of GLSs

The GLS analysis was performed as described previously [73,74]. The Chinese cabbage samples were extracted using 1 mL of 90% methanol solution with a tissue grinder (60 Hz, 1 min). The samples were incubated at 25 °C for 1 h, and then centrifuged at 8000 rpm for 10 min. An amount of 250 μL of Sephadex DEAE (Sigma-Aldrich, Shanghai, China) (1 g DEAE + 15 mL ddH_2_O) was diluted and transferred into a 1.5 mL centrifuge tube, and then centrifuged at 3200 rpm for 1 min. Then, 400 μL of crushed centrifuged sample supernatant was placed in a 1.5 mL centrifuge tube. Next, we mixed 900 μL of 90% methanol and centrifuged the mixture at 3200 rpm for 1 min. Finally, 210 μL of sulfatase (Sigma-Aldrich, Shanghai, China) (200 μL ddH_2_O + 10 μL Sulfatase) was poured into each tube, followed by thorough mixing and storage at 25 °C for 12 h in the dark. This was followed by centrifugation at 8000 rpm for 1 min and analysis of the purified samples using high-performance liquid chromatography (HPLC, Shimazu LC-2030 Plus, Kyoto, Japan). GLSs were separated using an CNW Athena C18-WP column (4.6 × 250 mm, 5 μm), and the column temperature was 37 °C. The sample (50 μL) was analyzed at the flow rate of 1.0 mL/min and the detection wavelength was 229 nm in HPLC. The specific elution included phase A: ultra-pure water and phase B: 100% methanol (Simark, Guangdong, China). The elution conditions were as follows: 0 min: A-B (98.5:1.5); 0–4 min: A-B (93:7); 4–10 min: A-B (75:25); 10–22 min: A-B (20:80); 22–25 min: A-B (10:90); 25–27 min: A-B (65:35); and 27–29 min: A-B (98.5:1.5). The determined GSLs were 4OH-I3M (4-hydroxy-indol-3-ylmethyl), I3M (indol-3-ylmethyl), 4MO-I3M (4-methoxy-indol-3-ylmethyl), and NMO-I3M (N-methoxy-indol-3-ylmethyl) with their retention time being 11.4, 13.4, 14, and 15.4 min, respectively. The composition and content of GLSs were determined based on retention time and peak area. Representative GLS chromatograms of Chinese cabbage are provided in Figure S3. The GLS content of all sample data of this study is available at Mendeley Data (http://dx.doi.org/10.17632/cxdhggmdcj.1, accessed on 3 September 2022).

### 4.7. Sample Pretreatment, Extraction, and Analysis of Carotenoids

The carotenoids were determined according to Lee [75]. In brief, 2 g of lyophilized cabbage leaves were added to 5 mL of carotenoid extract solutions (acetone:ethanol = 1:1, v:v) and mixed well, followed by extraction for 70 min and centrifugation at 12,000 rpm for 10 min. This process was repeated twice, combining the supernatant. Under dark conditions, the supernatant was placed in a round bottom flask and evaporated to dryness under reduced pressure at 35 °C, dissolved with 1 mL of ethyl acetate, and filtered through a 0.22 μm organic filter into a 1.5 mL brown sampling bottle for analysis using an Agilent 1260 Infinity II HPLC (Agilent Technologies, Waldbronn, Germany), and carotenoids were separated using a YMC C30 column (YMC, Kyoto, Japan; 250 × 4.6; 5 μm). Carotenoids were identified using the typical retention time of the standard compounds, including lycopene (Sigma-Aldrich, Saint Louis, MO, USA), zeaxanthin and lutein (Solarbio, Beijing, China), and α-carotene and β-carotene (Wako, Osaka, Japan). The carotenoids were identified and quantified according to the method used by Morris et al. [76]. Carotenoids in Chinese cabbage leaves were quantified according to the external standard curve method, and the standard was accurately diluted with ethyl acetate to concentrations of 0 μg/mL, 25 μg/mL, 50 μg/mL, 100 μg/mL, and 200 μg/mL. The solutions were detected using high-performance liquid chromatography and a standard curve was drawn. Carotenoid HPLC chromatograms are provided in Figure S4. The specific elution included phase A: acetonitrile: methanol = 3:1 (containing 0.01% butylated hydroxytoluene (BHT)); phase B: 100% methyl-tert-butyl ether (MTBE) (containing 0.01% BHT). The elution conditions were as follows: 0 min: A-B (95: 5); 0–10 min: A-B (95: 5); 10–19 min: A-B (86: 14); 19–29 min: A-B (75: 25); 29–54 min: A-B (50:50); 54–66 min: A-B (26:74); and 67 min: A-B (95:5). The mobile phase flow rate was 1 mL/min, and the detection wavelength was 450 nm; the column temperature was 35 °C.

### 4.8. Gene Expression Analysis

Total RNA from Chinese cabbage tissues was extracted using an RNA extraction kit (Tiangen Biotech, Xi’an, China) following the manufacturer’s instructions. RNA reverse transcription was performed using a HiScript III 1st Strand cDNA Synthesis Kit (+gDNA wiper) (Vazyme Biotech Co., Ltd., Nanjing, China). Quantitative reverse transcription PCR (qRT-PCR) was performed as previously described [77]. The iQ5.0 Bio-Rad iCycler thermocycler (Bio-Rad, Hercules, CA, USA) was utilized for qRT-PCR. The template cDNA (50 ng/μL) and the amplification program were as follows: 96 °C for 15 s, 95 °C for 30 s, 60 °C for 20 s, and 72 °C for 30 s; 40 cycles. The glyceraldehyde-3-phosphate dehydrogenase (GAPDH, GO0048316) gene was employed as the internal control [78]. The primers used are provided in Table S3. The specificities of all the primers were further confirmed using NCBI Primer BLAST (https://www.ncbi.nlm.nih.gov/tools/primer-blast/, accessed on 12 September 2022). The relative gene expression was determined using the 2^−ΔΔCT^ method [79].

### 4.9. Statistical Analysis

The results were used for an analysis of variance (ANOVA) performed with SPSS software (SPSS version 23.0, Chicago, IL, USA), and the Duncan test was used for statistical significance. The analyzed data were expressed as means ± standard error (SE) of three biological replications in all measured parameters. PCA was visualized using an online tool (https://www.bioinformatics.com.cn, accessed on 20 September 2022). A clustering correlation heatmap with signs was generated using OmicStudio tools available at (https://www.omicstudio.cn, accessed on 15 October 2022).

## 5. Conclusions

In this study, we revealed the wide variability in indolic GLS content and composition reflected in orange Chinese cabbage lines for the first time. In all the tested samples, the content of indolic GLSs in the inner leaf was relatively high, and the anticancer compound I3M increased rapidly when it was close to the core of the leaf head. The results of the GLS content investigation in non-edible organs showed that the content of indolic GLSs in flowers and seeds was significantly higher than that in stems and siliques. The expression levels of genes such as light signal, MEP metabolism, and carotenoid and GLS metabolism were basically consistent with the metabolite accumulation pattern. These results broaden the current understanding of the accumulation patterns of health-promoting substances in orange Chinese cabbage leaves.

## Data Availability

Data are contained within the article or the Supplementary Materials.

## Supplementary Materials

The following supporting information can be downloaded at: https://www.mdpi.com/article/10.3390/plants12112120/s1, Figure S1: Pie chart showing the proportions of different GLS components in 9 lines of Chinese cabbage (inner leaf, outer leaf, and root) at the S4 period; Figure S2: The correlation network diagram of the (A) high GLS (15S1094) and (B) low GLS (20S530) lines with biological properties, pigment, and GLS substances. The different asterisks represent Pearson correlation coefficients at * *p* < 0.05, ** *p* < 0.01, and *** *p* < 0.001; Figure S3: Representative HPLC chromatogram of GLS compounds from 14S837 Chinese cabbage lines. Peak: 1, 4OH-I3M; 2, I3M; 3, 4MO-I3M; and 4, NMO-I3M; Figure S4: HPLC chromatogram of standard carotenoid compounds. Peak: 1, lutein; 2, zeaxanthin; 3, α-carotene; 4, β-carotene; and 5, lycopene; Table S1: Comparison of agronomic traits of 9 Chinese cabbage lines; Table S2: Percentage of variance obtained for all principal component analyses (PC) of the four species of Chinese cabbage (14S23, 15S1094, 20S530, and 18BC6); Table S3: The primer sequences used for examination in this study.
