# Supplementary material for: The Accumulation of Health-Promoting Nutrients from Representative Organs across Multiple Developmental Stages in Orange Chinese Cabbage
Source: Plants (Basel). 2023 May 26;12(11):2120. doi: 10.3390/plants12112120 (PMC10255399; doi:10.3390/plants12112120)
Supplement: Supplementary file 1 [file plants-12-02120-s001.zip › Supplementary Figures.pdf]

Supplementary Figures

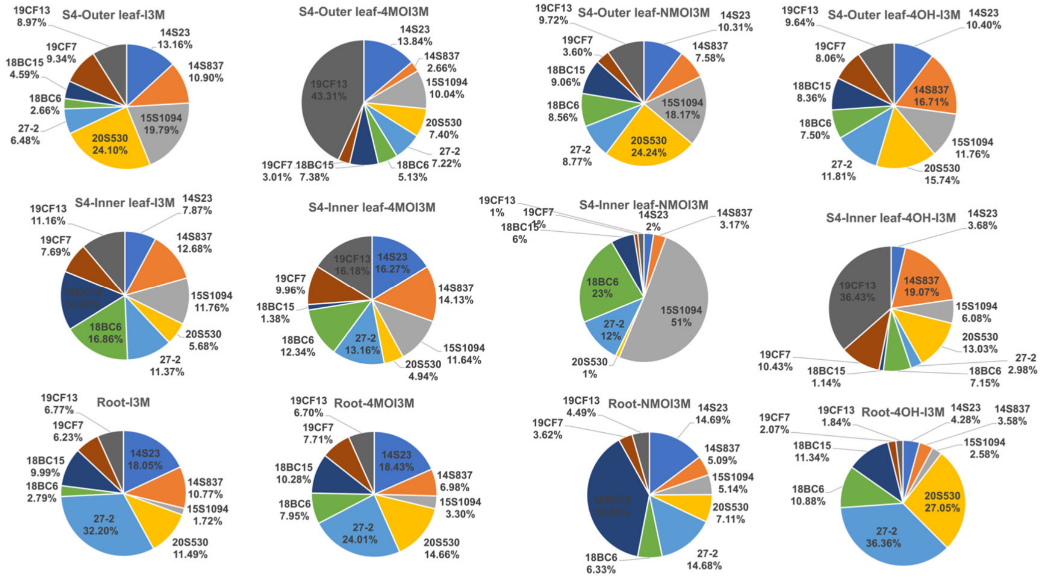

Figure S1. Pie chart showing the proportions of different GLS components in 9 lines of Chinese cabbage (inner leaf, outer leaf, root) at the S4 period.

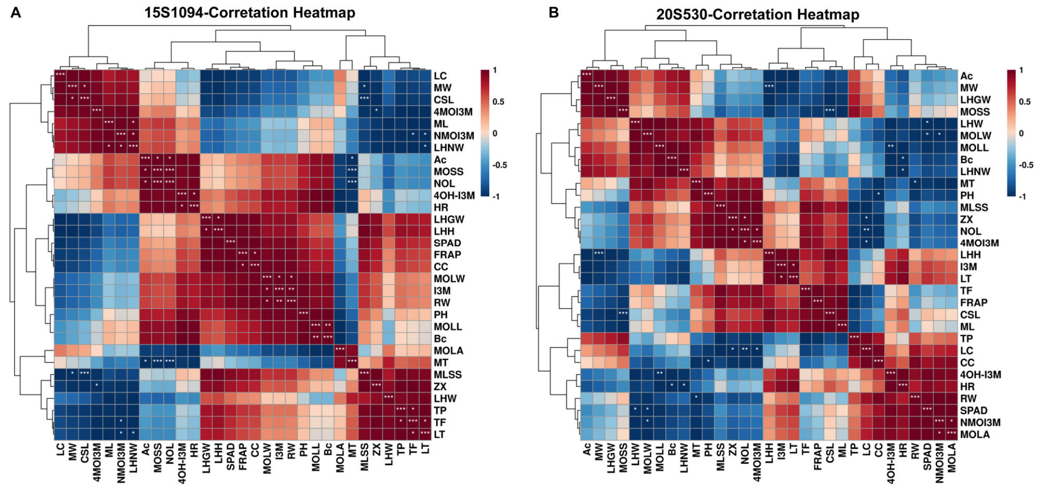

Figure S2. The correlation network diagram of the (A) High GLS (15S1094) and (B) low GLS (20S530) lines with biological properties, pigment, and GLS substance. The different asterisks represent Pearson correlation coefficients at *$P < 0.05$, **$P < 0.01$, and *** $P < 0.001$.

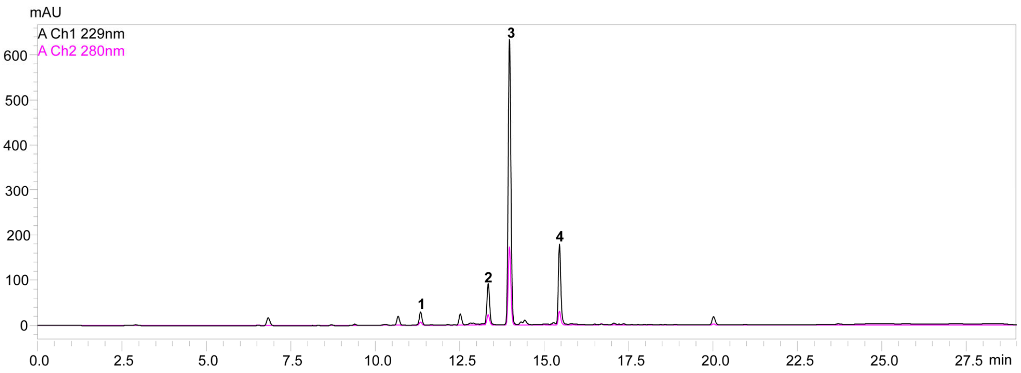

Figure S3. Representative HPLC chromatogram of GLS compounds from 14S837 Chinese cabbage lines. Peak: 1, 4OH-I3M; 2, I3M; 3, 4MO-I3M; 4, NMO-I3M.

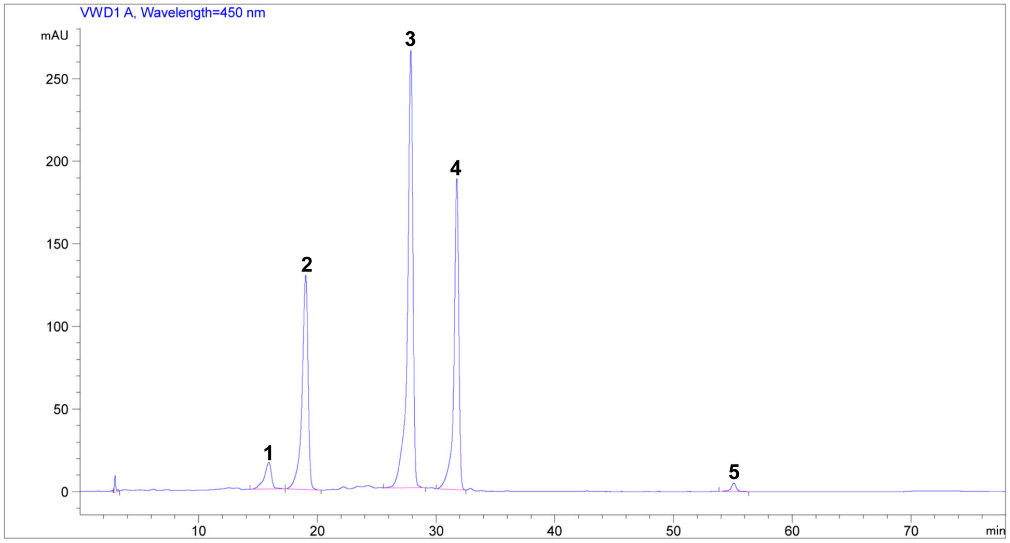

Figure S4. HPLC chromatogram of standard carotenoid compounds. Peak: 1, lutein; 2, zeaxanthin; 3, α-carotene; 4, β-carotene; 5, lycopene.
